# Molecularly Imprinted Polymer-Enhanced Electrochemical Sensor for Sensitive and Selective Captan Detection in Fruit Sample

**DOI:** 10.1021/acsomega.5c03951

**Published:** 2025-11-08

**Authors:** Melike Akan, Cigdem Kanbes-Dindar, Nazife Aslan, Bengi Uslu

**Affiliations:** † Polatlı Science and Arts Faculty, Chemistry Department, Ankara Hacı Bayram Veli University, 06900 Ankara, Turkey; ‡ Faculty of Pharmacy, Department of Analytical Chemistry, 37504 Ankara University, 06560 Ankara, Turkey

## Abstract

In this study, a molecularly imprinted polymer (MIP) was designed for the selective, rapid, and sensitive electrochemical detection of Captan, a pesticide. The sensor was fabricated by electropolymerizing *o*-phenylenediamine (*o*-PD) onto a glassy carbon electrode (GCE) in the presence of Captan as the template molecule, using cyclic voltammetry (CV). Differential pulse voltammetry (DPV) was used to facilitate template removal and reattachment, optimize experimental conditions, and evaluate sensor performance. A ferrocyanide/ferricyanide redox indicator monitored each stage of the experiment via DPV. The MIP@*o*-PD/GCE sensor demonstrated a linear response to Captan within the concentration range of 1 × 10^–14^ to 9 × 10^–14^ M under optimal conditions, making it suitable for quantifying Captan in apples. To verify its applicability, the sensor was successfully tested on apple samples. Additionally, potential interference was evaluated by comparing its response to KCl, CaCl_2_, MgCl_2_, Na_2_SO_4_, Ascorbic acid, glucose, and KNO_3_. The imprinting factor was used to demonstrate the selectivity of the proposed sensors using structurally similar compounds to Captan.

## Graphical abstract

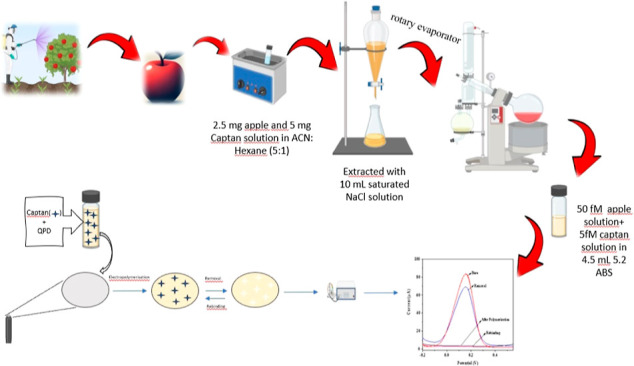

## 1 Introduction

Fungicides are crucial in modern agriculture, offering protection against fungal infections that can severely reduce crop yield and quality. These substances, whether chemical or biological, inhibit fungal growth and spore formation, protecting plants from diseases such as rust, blights, and mildews.^1^ However, their application must be carefully managed to minimize environmental impact and prevent resistance in fungal populations. Residues from fungicides can contaminate soil and groundwater, enter the food chain, and potentially affect the central nervous system of humans and livestock.^2,3^

Captan is a widely used contact fungicide, whose chemical structure is shown in Scheme 1 which targets fungal diseases on crops such as apples, strawberries and grapes, especially during the flowering period.^4,5^ This phenomenon disrupts fungal cellular processes, halting growth and reproduction. Captan is primarily surface-active and does not penetrate, deeply into plant tissues. Beyond agriculture, Captan is found in nonagricultural products like cosmetics (antibacterial soaps and shampoos), pharmaceuticals, paints, lacquers, adhesives, plasticizers, and rubber stabilizers.^6,7^

**Scheme 1:**
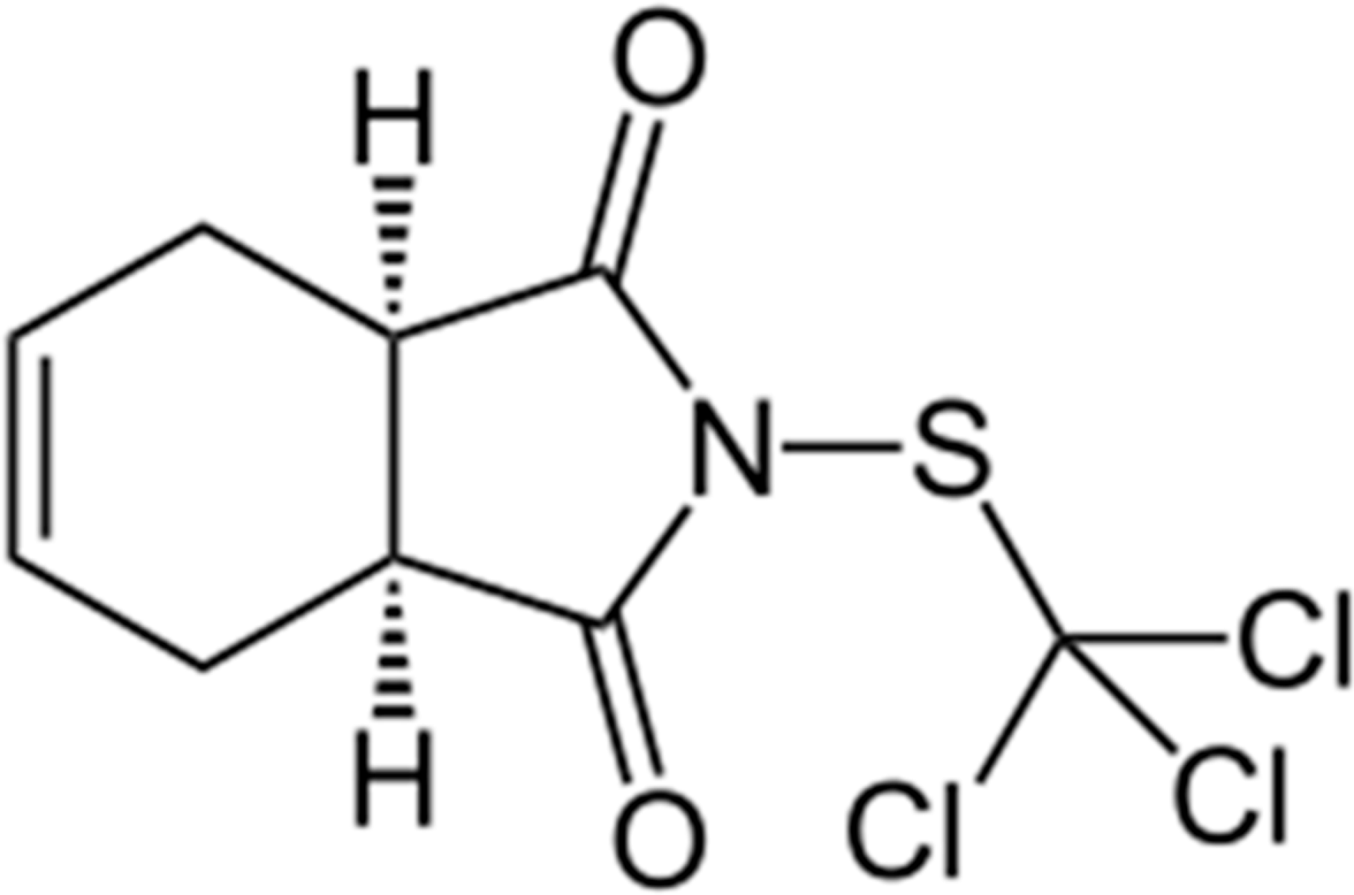
Chemical Structure of Captan

Although considered low in toxicity to humans, prolonged exposure to Captan can cause irritation and, at high doses, lead to nausea, diarrhea, or mild central nervous system effects.^8,9^ Rare cardiotoxic effects have also been documented, notably in individuals with low body weight, as reported by Gottzein et al. (2013).^10^ To ensure safety, Captan use is regulated using maximum residue limits (MRLs) established for food crops. For example, the EU sets MRLs for Captan in fruits like apples, pears, and cherries at 3 mg/kg, ensuring public health when good agricultural practices are followed.^11^

Detecting pesticide residues in food requires precise methods because of their trace-level presence. Techniques like gas chromatography–mass spectrometry^12–14^ (GC–MS), liquid chromatography–mass spectrometry^15,16^ (LC–MS), high-performance liquid chromatography^17–19^ (HPLC–MS), are commonly used. GC–MS, though sensitive, can face challenges with Captan’s thermal degradation during analysis, which can overestimate byproducts like tetrahydrophthalimide (THPI). LC–MS/MS offers an effective alternative, avoiding these complications and enabling accurate detection. Emerging technologies like surface-enhanced Raman spectroscopy (SERS) and advanced electrochemical sensors provide rapid, sensitive, and practical solutions for residue monitoring.^20–22^ Although these methods help ensure compliance with safety regulations and promote the sustainable use of fungicides in agriculture, they are not practical for directly detecting pesticides in actual samples.

Additionally, advanced electrochemical methods have attracted great attention due to their outstanding properties, such as accuracy, reliability, and time-saving.^23–27^ Moreover, electrochemical systems can be miniaturized and transformed into portable devices that facilitate high-precision on-site measurements.^28,29^ By leveraging selective electrodes and novel nanomaterials, electrochemical sensors can detect trace amounts of Captan in complex matrices such as soil, water, and agricultural products.^31–33^ This approach offers a more practical and efficient solution for monitoring Captan residues in various environmental and food samples, thereby supporting efforts to ensure safety and regulatory compliance in the agricultural sector.

Traditionally, most reported biosensors for Captan detection have relied on enzyme-based systems that exploit the inhibitory interactions between pesticides and enzyme activity.^39^ Although these biosensors demonstrate satisfactory performance, their utility is often compromised by inherent drawbacks such as high production costs, limited operational stability, enzyme denaturation, and strict environmental requirements. These limitations have prompted a shift toward nonenzymatic detection strategies, particularly electrochemical sensors based on molecularly imprinted polymers (MIPs), which exhibit superior stability, reproducibility, and cost-effectiveness.

MIPs are robust synthetic materials with highly specific molecular recognition sites.^34–36^ These sites are formed through the polymerization of functional monomers and cross-linkers in the presence of a template molecule. Following polymerization, the template is removed, leaving cavities designed to selectively rebind the original molecule based on its size, shape, and chemical properties. MIPs offer advantages such as stability under extreme pH conditions, cost-effectiveness, ease of preparation, and reusability. These attributes make them suitable for various applications, including chromatographic separation, solid-phase extraction, chemical sensing, and catalysis.^37–41^ Their ability to selectively recognize target molecules enhances their applicability in environmental and food safety monitoring, particularly for detecting contaminants like pesticides in complex matrices such as soil, water, and food products.^42^

In our study, a highly sensitive electrochemical sensor for Captan detection was designed and developed by integrating insights from previous research with the unique properties of MIPs. The sensor, designated as MIP@*o*-PD/GCE, was fabricated by electrodepositing *o*-PD and Captan onto a GCE using cyclic voltammetry. Experimental conditions were optimized, and the sensor’s analytical performance was thoroughly assessed. Its practical applicability was demonstrated through the successful detection of Captan residues in apple samples.

## 2 Experimental Section

### 2.1 Apparatus

Electrochemical experiments, including CV and DPV, were performed using a traditional three-electrode cell powered by an EMSTAT-4S potentiostat. The system was controlled using Palm Sense software on a personal computer. The standard three-electrode setup comprised a saturated Ag/AgCl (3 M KCl) reference electrode, a platinum plate counter electrode, and a GCE with a 3 mm diameter (BASi) as the working electrode.

The quantities used during the experiments were measured using a calibrated Shimadzu Instruments (Japan) precision balance, ensuring accurate material preparation. Solution pH levels were determined with a Mettler Toledo pH/ion meter (model S220, Switzerland), providing a precision of ±0.05 for optimal solution characterization. Removal and rebinding procedures during MIP fabrication and testing were facilitated using a Thermo-Shaker (Biosan TS 100), ensuring controlled and consistent experimental conditions.

The morphological properties of the MIP were investigated using a field emission gun scanning electron microscope (FEG-SEM), specifically the Hitachi SU-8700 model. This provided detailed imaging to evaluate the structure and uniformity of the synthesized MIP nanoparticles, which is essential for confirming their suitability for electrochemical sensor applications. Prior to analysis, the samples were attached to an aluminum stub using double-sided carbon tape and coated with a 10 nm layer of gold via a sputter coater.

### 2.2 Reagents

Captan (Pestenal, analytical standard, ≥98.0%) was obtained from Sigma-Aldrich, and a standard solution was prepared using acetonitrile and stored in a refrigerator to ensure stability. Additionally, Captan, analytical grade methanol (≥99.8%), sodium hydroxide (≥97%, pellets), sodium sulfate (≥99.0%, anhydrous, granular), sodium chloride (≥99.0%), acetic acid (≥99.7%), *o*-phenylenediamine (*o*-PD, ≥98.0%), sodium acetate trihydrate (≥99.0%), indole (≥99.0%), thiram (≥99.0%), ziram (≥99.0%), and tetraethylthiuram disulfide (≥99.0%) were procured from Sigma-Aldrich. High purity acetone and hexane solvents used for the extraction of Captan from the real sample were also purchased from Sigma-Aldrich. For electrochemical measurements, an acetate buffer solution (pH 5.2) was prepared using double-distilled water to ensure its purity and accuracy.

All prepared solutions were stored at 4 °C to maintain their stability throughout the study. Additionally, fresh apples were sourced from the local market for use in real sample applications, ensuring relevance to the intended environmental and food safety testing. This step is crucial for assessing the performance of the electrochemical sensor in a practical context, where simulating pesticide residues, such as Captan, may be present.

### 2.3 MIP and (Nonimprinted Polymer) NIP Based Electrochemical Sensors

Before initiating the electrochemical polymerization process, GCE was sonicated for 20 min in a solution consisting of methanol and distilled water mixed in a 1:1 ratio. The electrode surface was subsequently polished with an alumina slurry and a polishing pad, thoroughly rinsed with double-distilled water, and air-dried at room temperature.

To fabricate the molecularly imprinted polymer-modified electrode (MIP@*o*-PD/GCE), the GCE was immersed in a polymerization solution containing 5 mM *o*-PD, 0.5 mM Captan, and 0.1 M acetate buffer (pH 5.2). Electropolymerization (EP) was carried out by applying cyclic voltammetry over a potential range of −0.2 to 0.8 V for 10 cycles at a scan rate of 50 mV/s. A NIP sensor was prepared using the same procedure, excluding the Captan template from the polymerization mixture.

Following polymerization, the modified electrodes were rinsed with double-distilled water to remove unreacted components. Template removal from the MIP-modified electrode was achieved by immersing it in a 7:3 (v/v) mixture of 1 M hydrochloric acid and acetonitrile (ACN) for 15 min at room temperature. Subsequently, the electrode was incubated in Captan solutions of varying concentrations for 30 min at room temperature to facilitate analyte rebinding. Prior to each incubation step, the electrode surface was rinsed with distilled water for 30 s.

The electrochemical performance of the MIP-based sensor was evaluated in comparison with the NIP-based sensor, both of which were assessed under identical conditions. Electrochemical measurements were conducted in a solution containing 5 mM [Fe­(CN)_6_]^3–/4–^ and 0.1 M KCl. Also, error bars represent standard deviations from five independent measurements (*n* = 5).

### 2.4 Real Sample Application

The real-sample application of the developed MIP@*o*-PD/GCE sensor was tested using apples as the matrix. Four apples were obtained from the market, thoroughly washed, cut into pieces, and blended into a homogeneous mixture. A 2.5 g sample of this mixture was placed in a 100 mL beaker, followed by the addition of 5 mg of Captan and a 5:1 (v/v) acetone-to-hexane solvent mixture, resulting in a total volume of 25 mL. After stirring for 30 min, the sample was filtered using glass wool, transferred to a separatory funnel, and extracted with 10 mL saturated NaCl solution. After the organic phase was separated from the aqueous phase, 5 mL of hexane was added to the aqueous phase, and the extraction was repeated. The combined organic phases were filtered through the glass wool, dried over anhydrous Na_2_SO_4_ and concentrated on a rotary evaporator under reduced pressure until approximately 1 mL of solvent remained. Captan content in the apple samples was determined using the developed sensor.

## 3 Results and Discussion

### 3.1 Morphological Characterization of MIP@*o*-PD/GCE Sensor Platforms

Field emission gun scanning electron microscopy (FEG-SEM) and energy-dispersive X-ray spectroscopy (EDX) were employed to characterize and analyze the morphological features of the sensor surfaces. Figure 1 presents representative SEM images of the modified GCE at various stages: (A) bare GCE, (B) NIP, (C) Captan-MIP after template removal, (D) Captan-MIP, and (E) Captan-MIP following Captan rebinding.

**Figure 1:**
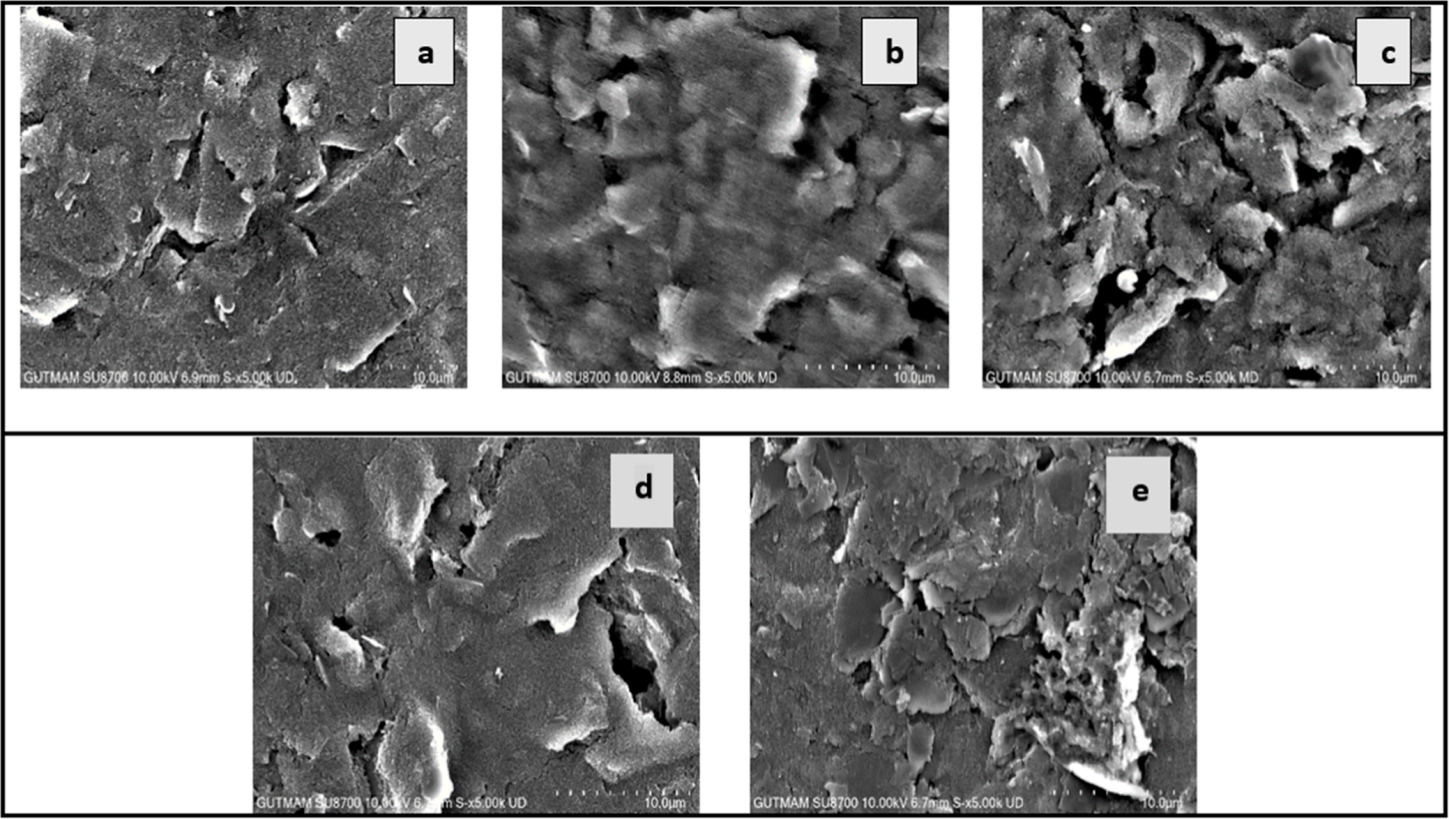
Field emission gun scanning electron microscopy (FEG-SEM) images of the GCE surface at different modification stages: (A) bare GCE, (B) NIP, (C), Captan-MIP after template removal (D) Captan-MIP, and (E) Captan-MIP following Captan rebinding.

As illustrated in Figure 1A, the bare GCE surface exhibits a spherical morphology, consistent with previous literature reports.^43^ Following modification with *o*-PD, the surface remains relatively uniform; however, the electropolymerized poly­(*o*-PD) film displays a rough and porous structure, as shown in Figure 1B. Upon Captan immobilization, Figure 1C reveals that the *o*-PD coating becomes less defined, with a noticeable increase in porosity and a less distinct surface topology. Subsequent removal of the Captan template (Figure 1D) results in a further increase in surface porosity and heterogeneity, thereby enhancing the accessibility of the molecular recognition sites within the imprinted polymer matrix.

Additionally, the energy dispersive spectroscopy (EDS) spectra in Figure S1 (Supporting Information file) offer further details on the composition of the surfaces of NIP, Captan-MIP, Captan-MIP (Captan removed), and Captan-MIP (Captan rebinding). When the EDX spectra of the NIP (Figure S1a) and MIP-Captan (Figure S1b) surfaces are compared, it is observed that the chlorine content increases from 1.6% to 2.3%, indicating that Captan was successfully bound to the polymer surface. The changes in chlorine content, shown in Figure S1c,d, where it decreases to 1.4% after Captan is removed and increases to 1.9% after rebinding, further support the conclusion that Captan was both removed from and reattached to the surface.

### 3.2 Electrochemical Characterization of MIP@*o*-PD/GCE Sensor

Electron transfer processes at the electrode surface and associated charge transfer resistance were evaluated using CV and electrochemical impedance spectroscopy (EIS). The electrochemical behavior of the modified GCE was examined in a 0.1 M KCl solution containing 5.0 mM [Fe­(CN)_6_]^3–/4–^ during the EP of 5 mM *o*-PD in the presence of 0.5 mM Captan. The sensor’s response was monitored at different fabrication stages, including EP, template removal, and analyte rebinding, as shown in Figure 2.

**Figure 2:**
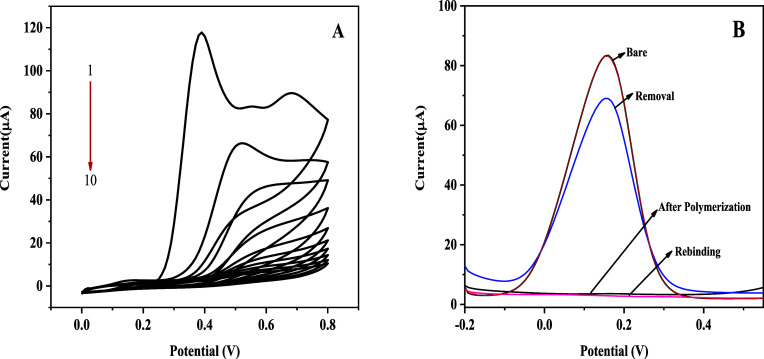
(A) Cyclic voltammograms and (B) differential pulse voltammograms recorded at different modification stages of the GCE: bare GCE (red), MIP@*o*-PD after EP (black), after template removal (blue), and after Captan rebinding in buffer (pink).

As depicted in Figure 2A, the bare GCE exhibited the highest peak current due to the absence of surface modifications, allowing unhindered electron transfer. Following EP, the electrode surface was coated with a polymeric film, which significantly impeded electron transfer, leading to the near-complete suppression of the [Fe­(CN)_6_]^3–/4–^ redox peak. Subsequent template removal resulted in the formation of specific recognition cavities within the polymer matrix, which partially restored electron transfer capability. However, the peak current remained lower than that observed for the bare GCE. Upon rebinding of Captan molecules to the imprinted cavities, the peak current of the [Fe­(CN)_6_]^3–/4–^ redox couple further decreased, indicating that the reoccupation of the recognition sites by the analyte partially obstructed electron diffusion pathways.

The alterations in charge transfer at the electrode surface were evaluated using Nyquist plots. EIS data (Figure 3B) revealed the lowest charge transfer resistance (*R*_ct_) for the bare GCE, measured at 59.3 Ω. Following EP, the *R*_ct_ significantly increased to 106.000 Ω, indicating that the polymeric film hindered electron transfer on the electrode surface. Upon removal of Captan molecules, the *R*_ct_ at the modified GCE decreased to 260 Ω, which, although higher than that of the bare GCE, suggests the formation of specific cavities that facilitated electron transfer. However, subsequent rebinding of Captan molecules to these cavities partially obstructed electron pathways, resulting in a marked increase in *R*_ct_ to 2.050 Ω. This increase confirms the selective recognition and binding of Captan within the imprinted polymer matrix.

**Figure 3:**
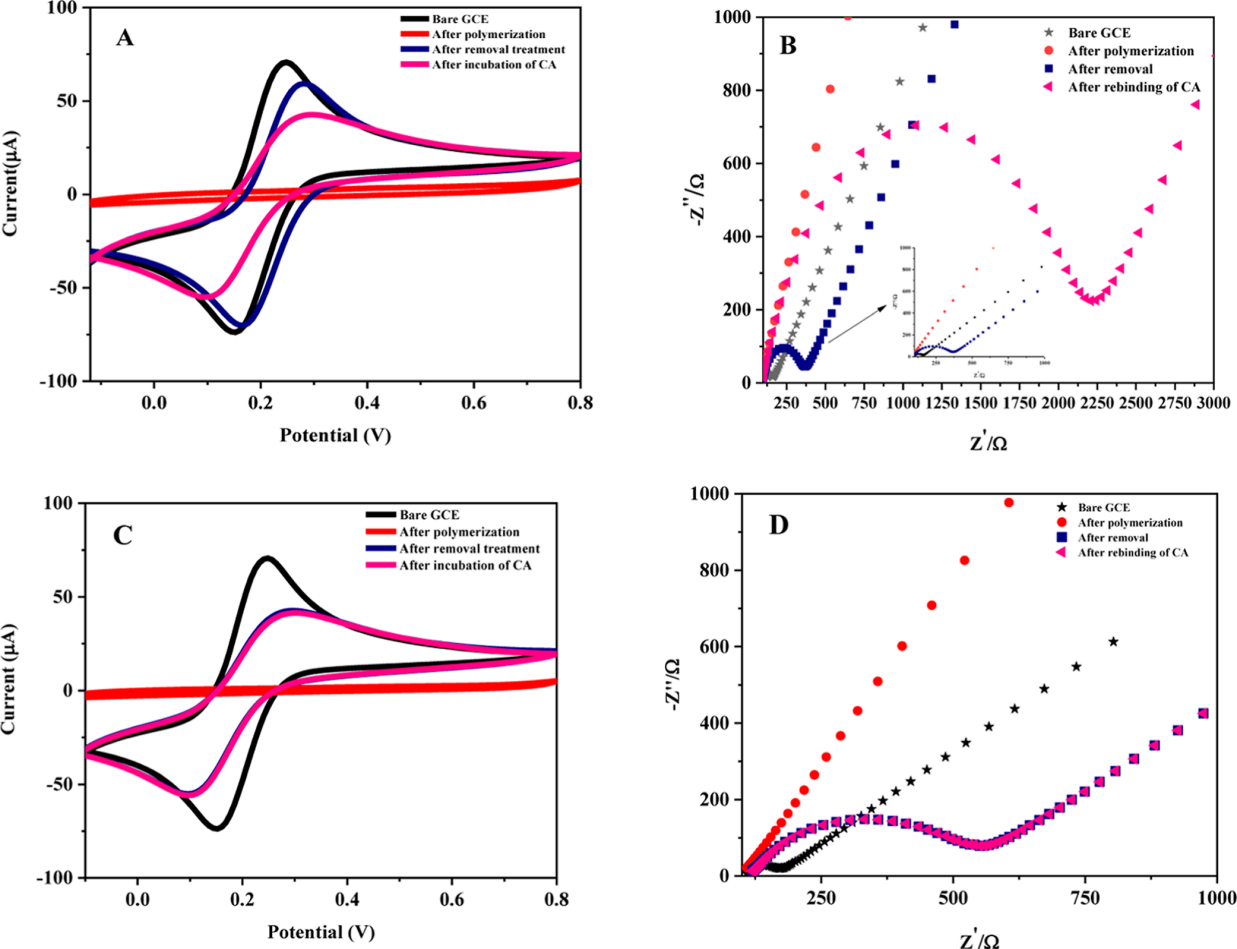
(A) Cyclic voltammograms (scan rate: 50 mV/s) (B) Nyquist plots of MIP/GCE (frequency range: 10^5^ to 10^–1^ Hz and amplitude: 10.0 mV) (C) NIP/GCE (D) Nyquist plots of NIP/GCE (in 0.1 M KCl solution containing 5.0 mM of [Fe (CN)_6_]^3–/4–^).

### 3.3 Optimization of Sensor Parameters

To construct a selective surface using *o*-PD, key parameters such as polymerization cycles, monomer-to-template ratio, extraction time, and rebinding time were systematically optimized to enhance the sensor’s durability and reliability. Each parameter was adjusted individually while keeping the others constant to isolate their effects.

The results showed that 10 polymerization cycles produced a fully coated polymer surface, yielding the highest postextraction current (Figure 4A). For the monomer-to-template ratio, four configurations (5:1, 10:1, 15:1, and 20:1) were tested, with the optimal peak current of 57 μA achieved at a 10:1 ratio (Figure 4B).

**Figure 4:**
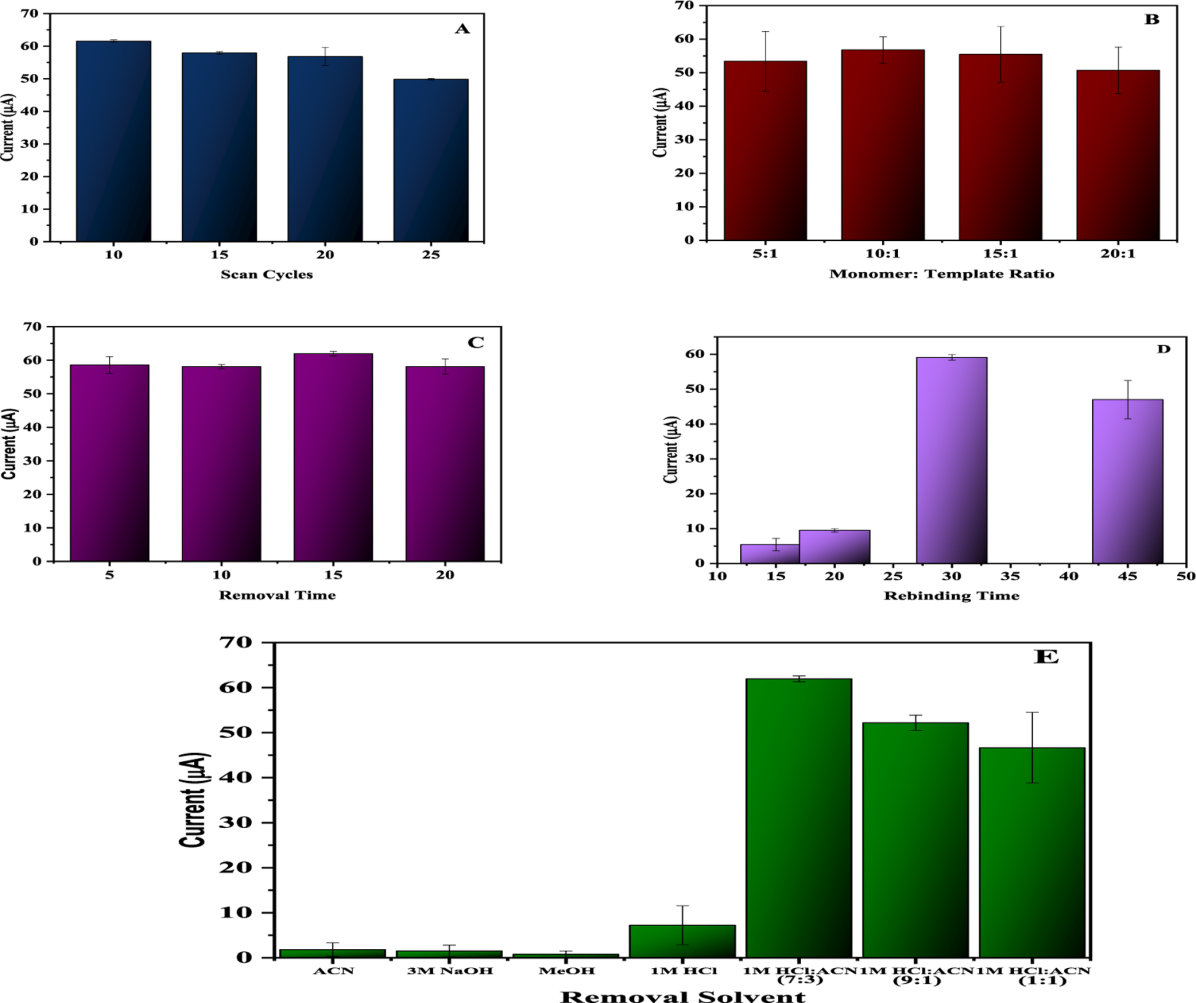
Optimization studies of EP cycles (A), monomer/template ratio (B), removal time (C), rebinding time (D), removal solvent (E), for development of MIP@*o*-PD/GCE.

Different solutions were tested for Captan extraction, including acetonitrile (ACN), 3 M NaOH, methanol, 1 M HCl, and mixtures of 1 M HCl and ACN in volume ratios of 7:3, 9:1, and 1:1. Among these, 1 M HCl/ACN (7:3) was the most effective solvent for removing Captan without damaging the polymer surface, with a 15 min extraction time found to be ideal (Figure 4C).

The optimal incubation time for rebinding Captan was determined using a 100 μM Captan solution, with 30 min yielding the best results (Figure 4D). Additionally, 1 M HCl/ACN (7:3) provided the highest reproducibility among the experimental conditions (Figure 4E). These carefully optimized parameters ensured that the MIP-based electrochemical sensor achieved excellent sensitivity, selectivity, and consistency for Captan detection. These systematically optimized parameters collectively contributed to the development of a highly sensitive, selective, and reliable MIP-based electrochemical sensor for the detection of Captan.

### 3.4 Assessment of Interference Effects

The selectivity of the MIP@*o*-PD/GCE sensor was evaluated in the presence of potential interfering substances commonly found in fruit matrices, including diazinon, ascorbic acid, glucose, chloride (Cl^–^), sulfate (SO_4_^2–^), magnesium (Mg^2+^), and nitrate (NO_3_^–^) ions. DPV was employed to assess the sensor’s response, and recovery values were calculated to determine the extent of interference. The recovery rates for Captan detection using the MIP@*o*-PD/GCE sensor ranged from 92.38% to 104.9%, indicating negligible interference from the tested species. These results demonstrate the sensor’s high selectivity and confirm its reliability for accurate Captan detection in complex sample matrices (Figure 5).

**Figure 5:**
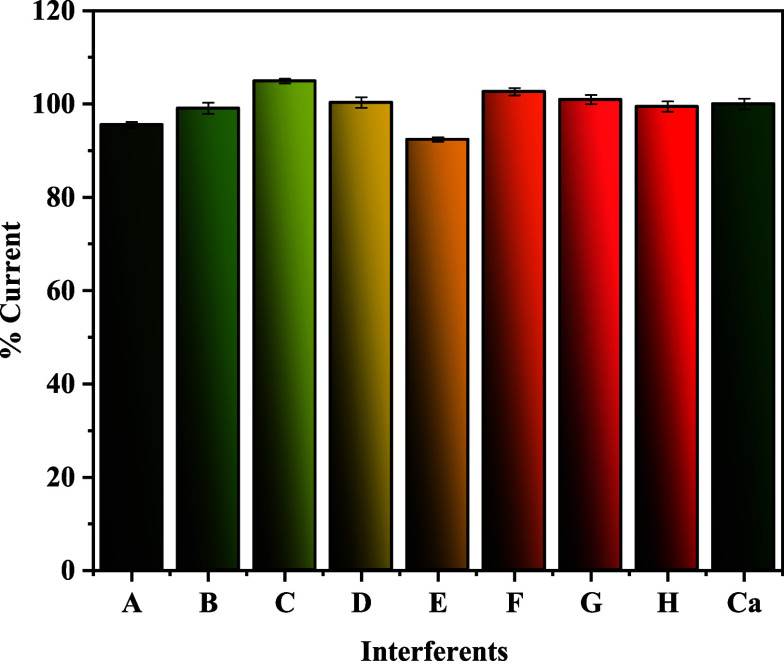
DPV signal of Captan in the presence of various interfering agents. (A) CaCl_2_, (B) KCl, (C) Na_2_SO_4_, (D) glucose, (E) MgCl_2_, (F) KNO_3_, (G) ascorbic acid, (H) diazinon (Captan/interferences molar ratio 1:1).

### 3.5 Selectivity of the Sensor

The selectivity coefficient (*k*) is a key indicator of the performance of MIPs, reflecting the efficiency of the formation of selective recognition sites within the polymer matrix. In this study, indole, a compound structurally similar to Captan, was employed as a model interferent to assess the selectivity of the developed MIP-based sensor. The selectivity coefficient (*k*) was calculated by comparing the current response (Δ*I*) of MIP- and NIP-modified electrodes following the rebinding of 1 × 10^–8^ M concentrations of Captan and the model compound such as indol, thiram, ziram and, tetraethyl thiuram disulfide­(TTD).^45^

The MIP sensor exhibited a *k* value of 8.45, in contrast to the NIP sensor, which demonstrated a much lower *k* value of 1.13. Furthermore, the relative selectivity coefficient (*k*′), defined as the ratio of *k* values for MIP to NIP, was determined to be 7.48. Furthermore, similar trends were observed for ziram, thiram and tetraethylthiuram disulfide, with *k*′ values of 5.40, 5.30 and 5.85, respectively. These results clearly indicate that the MIP sensor possesses a strong preferential binding affinity for Captan over structurally related compounds, as evidenced by *k*′ values significantly greater than 1.0 (Table S1).

To further validate the sensor’s selectivity, competitive binding experiments were conducted in the presence of both Captan and indole. As shown in Figure 6, there was no significant difference between the sensor responses in solutions containing Captan alone and those containing both Captan and indole. This outcome confirms that the sensor maintains high selectivity for Captan even in the presence of potentially interfering species, thus demonstrating its suitability for application in complex sample matrices.

**Figure 6:**
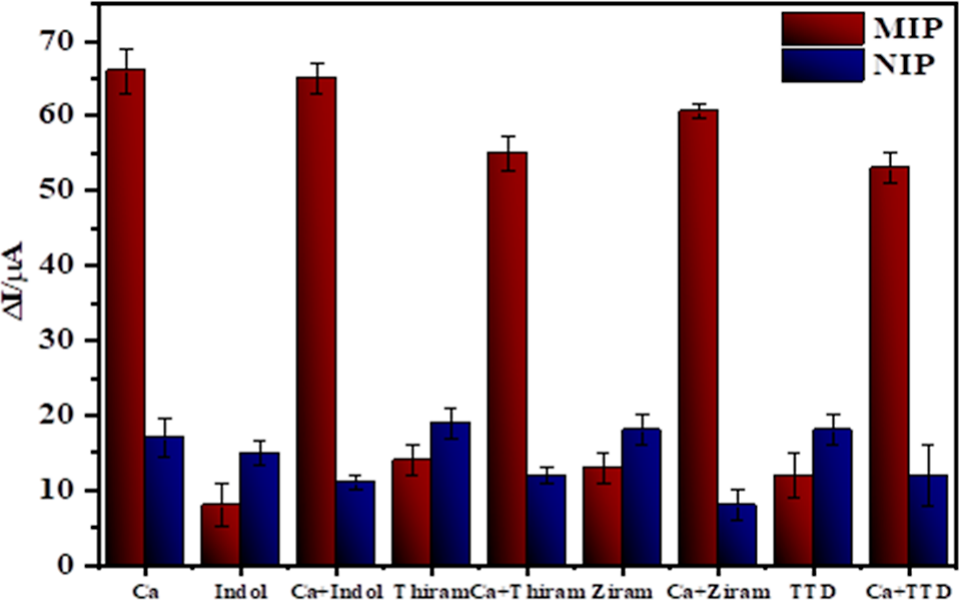
Selectivity of MIP@*o*-PD-CA/GCE sensor for Captan.

### 3.6 Electroanalytical Performance of the Sensor

The electroanalytical performance of the MIP@*o*-PD-CA/GCE sensor was evaluated using a standard solution via the DPV method. To evaluate the binding of different Captan concentrations to specific recognition sites, an indirect method was used. This involved calculating the difference in peak current for a 5 mM [Fe­(CN)_6_]^3–/4–^ solution acting as a redox probe, rather than performing direct electrochemical measurements. The corresponding voltammograms are depicted in Figure 7A. Figure 7B shows a linear relationship between the sensor response and Captan concentration over a specific range of 1.0 × 10^–14^ M to 9 × 10^–14^ M. No linear response was detected when the same procedures were applied to a nonimprinted polymer with no specific binding sites. The comparison results showed that the MIP@*o*-PD/GCE exhibits exceptional selectivity and a strong binding affinity for Captan. The regression equation for the change in current (Δ*I*) was derived from these measurements as Δ*I* = 4 × 10^12^ × *C* + 0.0276, with a correlation coefficient of *r* = 0.9929. Moreover, since the ultimate goal of the sensor system was the determination of Captan from apple samples, the calibration curves obtained in aqueous and matrix-based systems (Cal 1 and Cal 2) were compared under the same experimental conditions, as shown in Figure S2. The results indicated that there was no statistically significant difference between the two calibration plots, suggesting negligible matrix effect in this case.

**Figure 7:**
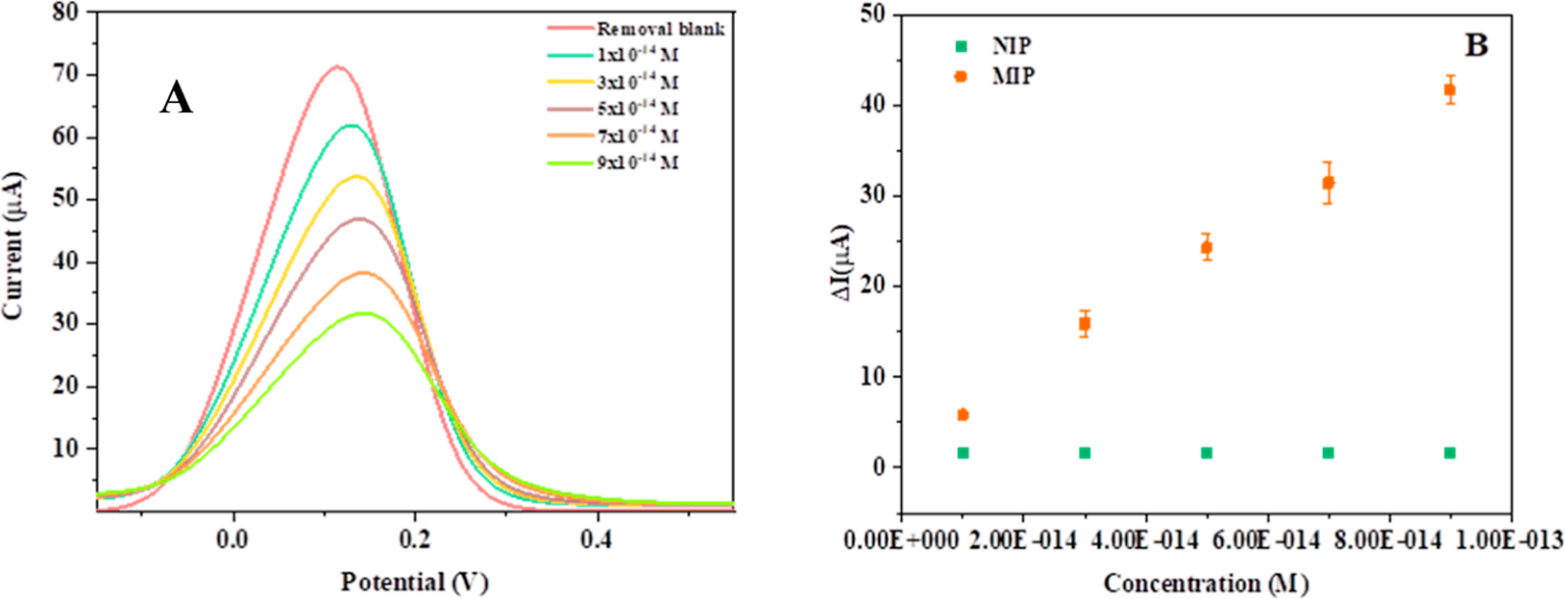
DP voltammograms of MIP@*o*-PD/GCE with different Captan concentrations in buffer (A) solution. Calibration curve of Captan with MIP@*o*-PD/GCE and (B) standard solution.

The validation parameters of the developed method, such as the linear working ranges, limit of detection (LOD), and limit of quantification (LOQ), were thoroughly evaluated. The observed variations in both the potential and peak current over daily and multiday intervals emphasize the sensitivity of the proposed method and its ability to maintain stable performance over time. The equations 3 *s*/*m* and 10 *s*/*m* were used to calculate the LOD and LOQ, where “*s*” represents the standard deviation of the response and “*m*” refers to the slope of the calibration curve.^44,45^ The LOD and LOQ values listed in Table 1 clearly illustrate the method’s sensitivity. Moreover, reproducibility was assessed by fabricating and analyzing ten independently prepared sensors under identical conditions. The peak currents measured after the polymerization, removal, and rebinding steps were recorded for each electrode. The relative standard deviation (RSD %) of the peak current responses was found to be 3.21% after polymerization, 1.88% after template removal, 3.71% after template rebinding Table S2.

**Table 1:** Validation Parameters of the Developed Sensor *for Captan on MIP-Based GCE*

parameters	MIP@*o*-PD/GCE
linear working range (M)	1 × 10^–14^ to 9 × 10^–14^
slope (μA/M)	4 × 10^12^
intercept (μA)	0.0276
correlation coefficient (*r*)	0.9929
LOD (M)	1.97 × 10^–15^
LOQ (M)	6.56 × 10^–15^
intraday precision of peak current (relative standard deviation, RSD %)	1.42
interday precision of peak current (RSD %)	7.31

Table 2 summarizes the analytical performance characteristics of various electrochemical (bio)­sensors for Captan determination, detailing their linear working ranges, LOD and real sample applications. The MIP@*o*-PD/GCE sensor stands out with superior analytical performance, especially due to its significantly broader linear working range and highly sensitive detection limit.

**Table 2:** Comparison of Analytical Performance Parameters of the MIP@*o*-PD/GCE Sensor with Other Previously Reported Electrochemical Captan Sensors

electrode modification	detection method	linear range (μM)	LOD (nM)	sample	ref
Pt/CeO_2_/chitosan	LSV	0.005–0.030	0.085	ground water	^31^
Au/APTES/GDA/GST	CV	0.83–53		tap water, drinking water, Milli-Q water	^29^
SPCE-Chi-GST	LSV	0–4.18, 8.32–49.9	670		^30^
Pt/ZnO/AChE	CV	0.05–25.0	107	apple	^44^
MIP@*o*-PD/GCE	DPV	1.0 × 10^–8^ to 9.0 × 10^–8^	1.97 × 10^–6^ (0.07 ng/kg)	apple	*this work*

### 3.7 Application of MIP@*o*-PD/GCE to Apple Samples

To evaluate the practical applicability of the developed MIP@*o*-PD/GCE sensor, Captan detection was conducted in spiked apple samples using the standard addition method. Calibration solutions were prepared from intermediate dilutions of the Captan stock solution to achieve final concentrations of 1.0 × 10^–14^, 5.0 × 10^–14^, and 9.0 × 10^–14^ M. For each concentration, 500 μL aliquots of Captan-spiked apple samples were analyzed under optimized experimental conditions.

As summarized in Table 3, the recovery rates for Captan in apple samples were determined to be 100.2%, 100.8%, and 99.1% for the respective concentrations. The relative standard deviation (RSD) values were 1.34%, 1.02%, and 1.20%, respectively, demonstrating high precision and repeatability of the sensor.

**Table 3:** Results of Real Sample Analyses (*N* = 5) (Apple Samples in 0.05 M (pH 5.2) of Acetic Acid/Acetate BS, *E*_start_: −0.2 V, *E*_finish_: 0.8 V, Step Amplitude: 8 mV, Pulse Amplitude: 20 mV, Scan Rate: 50 mV/s)^a^

Captan added (M)	Captan found (M)	recovery (%)	RSD (%)	*t*_experimental_*
1.0 × 10^–14^	1.002 × 10^–14^	100.2	1.34	2.47
5.0 × 10^–14^	5.042 × 10^–14^	100.8	1.02	1.99
9.0 × 10^–14^	8.922 × 10^–14^	99.1	1.20	–2.45

a Each value is the average of five experiments. RSD %: relative standard deviation %, * *t*_critical_ is 2.78 for 4 degrees of freedom at the 95% confidence level.

To further validate the method’s accuracy, Student’s *t*-test was applied to compare the measured Captan concentrations with the theoretical values. The calculated *t*-values (*t*_experimental_) for the 1.0 × 10^–14^ M and 5.0 × 10^–14^ M concentrations were 2.47 and 1.99, respectively, both of which were below the critical *t*-value (*t*_critical_) at the 95% confidence level. Similarly, for the 9.0 × 10^–14^ M concentration, the *t*_experimental_ value exceeded—*t*_critical_, indicating no statistically significant difference between the added and detected concentrations of Captan in all cases.

These findings confirm that the developed sensor exhibits excellent analytical performance in real sample matrices, offering high sensitivity, accuracy, and reproducibility for the detection of Captan residues in fruit-based food products.

## 4 Conclusion

Electrochemical sensing platforms based on MIPs present a promising alternative to conventional analytical techniques due to their cost-effectiveness, operational simplicity, environmental compatibility, use of nontoxic reagents, and low energy requirements. In this study, a highly selective and sensitive electrochemical sensor, MIP@*o*-PD/GCE, was successfully developed for the detection of Captan pesticide.

The sensor was fabricated via the EP of *o*-PD in the presence of Captan on a glassy carbon electrode, forming selective recognition sites tailored to the target analyte. The sensor fabrication and performance were monitored using ferrocyanide/ferricyanide redox probes and characterized through DPV and CV techniques.

The developed sensor demonstrated excellent analytical performance in real sample applications, achieving high recovery rates ranging from 99.1% to 100.8% in spiked apple samples. Through systematic optimization of fabrication parameters, the sensor exhibited superior detection and quantification limits, reproducibility, and reliability compared to previously reported Captan sensors.^31,46^ To date, no electrochemical (bio)­sensor has been reported with both a linear detection range and a detection limit at the picomolar level for Captan*.*

Thus, the MIP@*o*-PD-CA/GCE sensor offers a practical and efficient tool for the trace-level detection of Captan residues in fruit samples, contributing significantly to food safety assurance and regulatory compliance.

## Supplementary Material



## Data Availability

The data underlying this study are available in the published article and its Supporting Information.

## Notes

SEM images of (a) NIP, (b) Captan-MIP, (c) Captan-MIP (Captan removed), (d) Captan-MIP (Captan rebinded) GCE surface (Figure S1) (PDF)
