# Supplementary material for: Molecularly Imprinted Polymer-Enhanced Electrochemical Sensor for Sensitive and Selective Captan Detection in Fruit Sample
Source: ACS Omega. 2025 Nov 8;10(45):53897–906. doi: 10.1021/acsomega.5c03951 (PMC12631419; doi:10.1021/acsomega.5c03951)
Supplement: Supplementary file 1 [file ao5c03951_si_001.pdf]

**Supporting information**

# Molecularly Imprinted Polymer-Enhanced Electrochemical Sensor for Sensitive and Selective Captan Detection in Fruit Sample

Melike AKAN[1], Cigdem Kanbes-Dindar[1,2*,] Nazife ASLAN[1], Bengi USLU[2]

[1]Ankara Hacı Bayram Veli University, Polatlı Science and Arts Faculty, Chemistry Department, 06900, Ankara, Türkiye

[2]Ankara University, Faculty of Pharmacy, Department of Analytical Chemistry, 06560, Ankara, Türkiye

*Corresponding author e-mail: ckanbes@gmail.com, cigdem.dindar@hbv.com

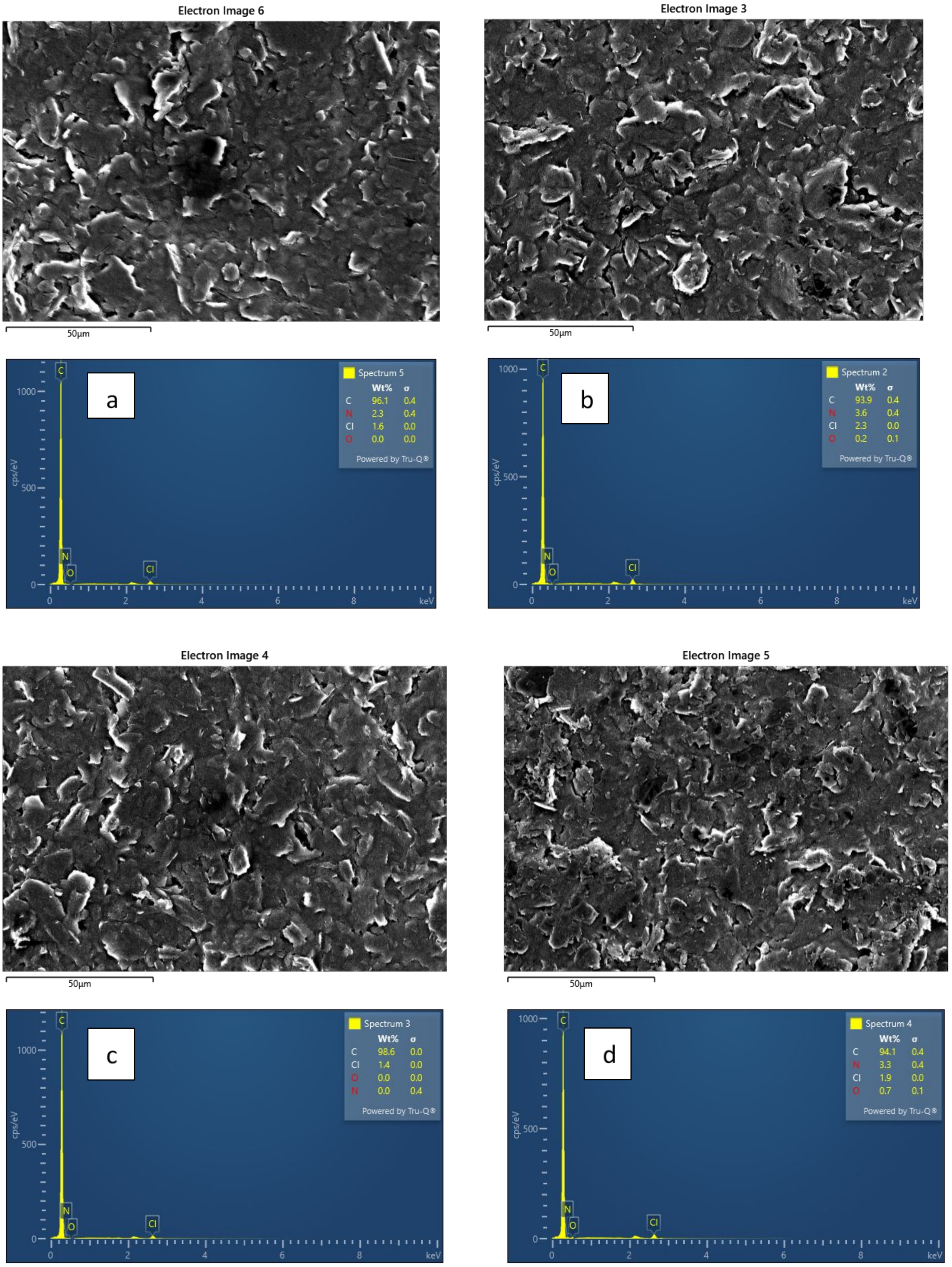


**Figure S1.** SEM images of (a) NIP, (b) Captan-MIP, (c) Captan-MIP (Captan removed), (d) Captan-MIP (Captan rebinded) GCE surface.

**Figure S2.**

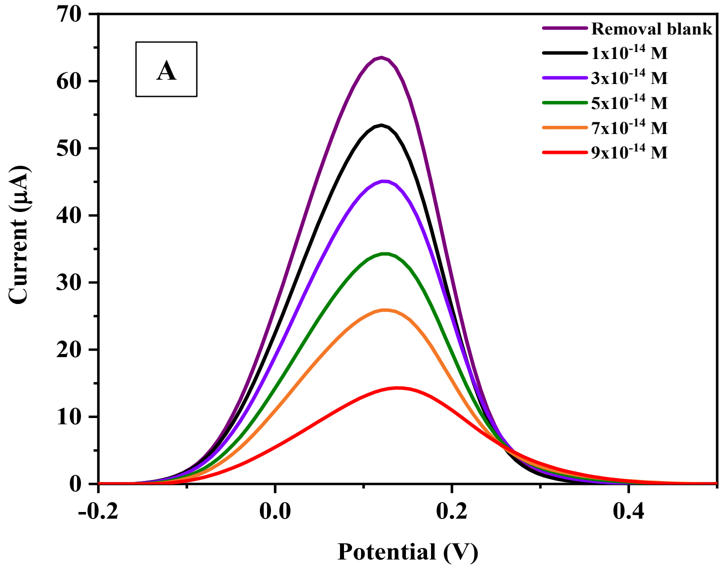


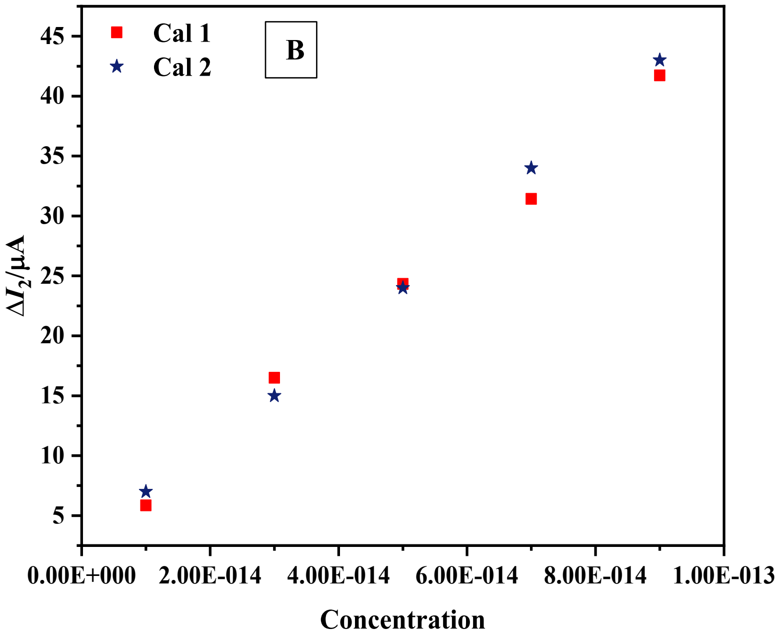


**Figure S2.** DP voltammograms of MIP@o-PD/GCE with different *Captan* concentrations in buffer solutions including acetone/hexane(1:1)matrix (A). Calibration curve of *Captan* with MIP@o-PD/GCE buffer solutions including acetone/hexane(1:1)matrix. Cal1: standard buffer solution calibration and Cal 2: standard buffer solution calibration including acetone/hexane(1:1)matrix (B)

**Table S1.** Selectivity of o-PD/Captan/MIP-GCE for *Captan*

| | **Δ*I*(μA$^{-1}$)** | **IF$_{(MIP)}$** | **Δ*I*(μA$^{-1}$)** | **IF$_{(NIP)}$** | **IF(k') $_{(MIP/NIP)}$** |
|---|---|---|---|---|---|
| **Captan** | 660 | - | 170 | - | - |
| **Indol** | 778 | 8.45 | 150 | 1.13 | 7.48 |
| **Ziram** | 130 | 5.07 | 180 | 0.94 | 5.40 |
| **Thiram** | 140 | 4.71 | 190 | 0.89 | 5.30 |
| **Tetraethyl thiuram disulfide** | 120 | 5.5 | 180 | 0.94 | 5.85 |

**Table S2.** Reproducibility of o-PD/Captan/MIP-GCE for *Captan*

| **Number of electrodes** | **After polymerization (nA)** | **Removal (mA)** | **Rebinding (nA)** |
|---|---|---|---|
| **1** | *7.0* | *0.611* | *2.21* |
| **2** | *6.8* | *0.598* | *2.30* |
| **3** | *6.5* | *0.604* | *2.10* |
| **4** | *7.0* | *0.634* | *2.21* |
| **5** | *6.8* | *0.614* | *2.30* |
| **6** | *6.5* | *0.619* | *2.10* |
| **7** | *7.0* | *0.599* | *2.21* |
| **8** | *6.8* | *0.626* | *2.30* |
| **9** | *6.5* | *0.620* | *2.10* |
| **10** | *7.0* | *0.610* | *2.21* |
| **Average** | *6.79* | *0.610* | *2.20* |
| **Standard deviation** | 0.21 | 0.011 | 0.08 |
| **Inter-day precision of peak current (RSD%)** | **3.21** | **1.88** | **3.71** |
